# Nucleus size and its effect on nucleosome stability in living cells

**DOI:** 10.1016/j.bpj.2022.09.019

**Published:** 2022-09-21

**Authors:** Artem K. Efremov, Ladislav Hovan, Jie Yan

**Affiliations:** 1 Institute of Systems and Physical Biology, Shenzhen Bay Laboratory, Shenzhen, China; 2 Mechanobiology Institute, National University of Singapore, Singapore, Singapore; 3 School of Pharmaceutical Sciences, University of Geneva, Geneva, Switzerland

## Abstract

DNA architectural proteins play a major role in organization of chromosomal DNA in living cells by packaging it into chromatin, whose spatial conformation is determined by an intricate interplay between the DNA-binding properties of architectural proteins and physical constraints applied to the DNA by a tight nuclear space. Yet, the exact effects of the nucleus size on DNA-protein interactions and chromatin structure currently remain obscure. Furthermore, there is even no clear understanding of molecular mechanisms responsible for the nucleus size regulation in living cells. To find answers to these questions, we developed a general theoretical framework based on a combination of polymer field theory and transfer-matrix calculations, which showed that the nucleus size is mainly determined by the difference between the surface tensions of the nuclear envelope and the endoplasmic reticulum membrane as well as the osmotic pressure exerted by cytosolic macromolecules on the nucleus. In addition, the model demonstrated that the cell nucleus functions as a piezoelectric element, changing its electrostatic potential in a size-dependent manner. This effect has been found to have a profound impact on stability of nucleosomes, revealing a previously unknown link between the nucleus size and chromatin structure. Overall, our study provides new insights into the molecular mechanisms responsible for regulation of the nucleus size, as well as the potential role of nuclear organization in shaping the cell response to environmental cues.

## Significance

The nucleus plays a central role in the life of eukaryotic cells, providing the highest level of control of intracellular processes. Depending on the stage of the cell cycle and/or surrounding environment, the size of the cell nucleus may undergo changes that are believed to cause chromatin reorganization, affecting gene transcription. However, there is currently no clear understanding of the molecular mechanisms that may be responsible for such regulation, the exact effect of which on chromatin structure remains unclear. In this study, by developing an advanced computational approach, we explore these problems from a physical perspective, revealing previously unknown mechanisms contributing to organization of the cell nucleus and chromatin.

## Introduction

Homeostasis and biological functioning of living cells rely on sophisticated synergistic cooperation between multiple molecular subsystems that must coexist with each other in a tight and highly crowded cellular space. This makes the problem of space allocation to each of the cell components one of the most important for intracellular organization, especially taking into account that many of the subcellular systems have very different requirements to the surrounding microenvironment needed for their proper operation. However, at the present time, there is still no clear understanding of molecular mechanisms responsible for size regulation of most of the cellular organelles, including even the major ones, such as the cell nucleus (1, 2, 3, 4, 5). Indeed, although many models have been developed in the past with varying degrees of detail to describe the regulation of cell volume (6, 7, 8), there is practically no similar analog to address the problem of the nucleus size control.

Previously, several cell components have been proposed as potential key factors contributing to the regulation of the cell nucleus size. These include cytosolic macromolecules and chromosomal DNA, which exert external and internal pressure on the nuclear envelope (NE), respectively (9, 10, 11, 12); lamins that drive cell-cycle-dependent nucleus growth in metazoan cells by polymerizing into the dense lamina network inlaying the NE (13, 14, 15, 16), and endoplasmic reticulum (ER)-binding proteins that have been found to retard nuclear expansion (1,17). Finally, it has been suggested that interactions between the actin cytoskeleton and NE proteins, such as nesprins, may also affect the shape of the cell nucleus (18). Although the major cellular components potentially influencing the nucleus size have been identified, a theoretical model that could integrate most of them to better understand the molecular mechanisms responsible for the nucleus size regulation has not yet been developed. As a result, such a well-known experimental phenomenon as the correlation between the cell and nucleus volumes, which is observed in many eukaryotic cells (19, 20, 21, 22), currently remains obscure.

In addition, the nucleus size has been previously shown to strongly correlate with chromatin structure in living cells (23, 24, 25, 26), suggesting that it may have a major impact on a number of cellular processes. Yet, the physical mechanisms underlying this link between the nucleus size and chromatin structure are not well understood. This makes it hard to fully comprehend potential effects of various extracellular and intracellular factors on the nuclear organization, as well as to interpret complex and often contradictory experimental data. For example, while some in vitro studies of isolated nuclei indicate that chromosomal DNA plays a central role in determining physical properties of the nucleus (12), others demonstrate that confined DNA does not affect the nucleus size, but rather sets its minimum possible value (9)—an observation that is supported by multiple in vivo studies showing no correlation between the size of the cell nucleus and its DNA content (21,27, 28, 29).

The main difficulty in understanding the role of the nucleus size in shaping the chromatin structure comes from the fact that the latter is predominantly determined by a tight interplay between several key factors, whose exact contributions cannot be easily quantified. Indeed, existing mathematical models are usually based on a heavily coarse-grained description of chromatin, using extended chromatin fiber segments as elementary modeled units (30, 31, 32, 33, 34, 35, 36, 37, 38, 39, 40, 41). This makes it impossible to take into consideration behavior of individual nucleoprotein complexes, such as their formation and disassembly, which are the main molecular processes responsible for chromatin organization. Furthermore, due to computational limitations, existing theoretical models do not take into account the major physical force that affects chromatin structure—electrostatic DNA-DNA and protein-DNA interactions. As a result, the role of the intracellular ionic environment in chromatin organization still remains poorly understood despite experimental studies showing that it plays an important role in shaping the chromatin structure (23,24,42, 43, 44, 45, 46). Thus, to accurately describe the correlation between the nucleus size, ionic environment, chromatin structure, and physicochemical properties of individual nucleoprotein complexes, development of an alternative theoretical approach is required.

In this study, by utilizing elements of polymer field theory and statistical physics, we have constructed such an integrated theoretical framework that allows one to address the above problems and predict the size of the cell nucleus, as well as the conformation of chromosomal DNA, by taking into account the dynamic nature of nucleoprotein complexes and the main physical forces responsible for nuclear organization. As a result of model calculations, it has been found that the nucleus size in the general case is predominantly determined by a tug-of-war between the osmotic pressure exerted by cytosolic macromolecules on the NE and the difference between the surface tensions of the NE and ER membrane. In addition, the model showed the existence of a previously unknown physical link between chromatin structure and the nucleus size, revealing a new potential molecular pathway, which may contribute to cell mechanosensing.

## Theoretical framework

In this work, we used the previously developed theoretical framework based on transfer-matrix calculations that allows one to compute the grand partition function of DNA in the presence of DNA-binding proteins (47, 48, 49), extending it to include in consideration cellular components and physical forces discussed in the introduction. Whereas all mathematical details can be found in Online Appendices A–M, here we will only outline the central idea of the study and the main model assumptions.

**Figure 1:**
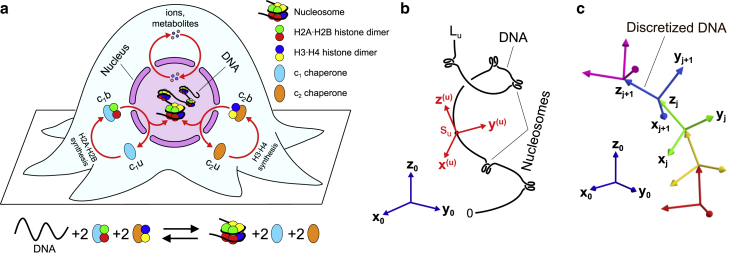
Semiflexible polymer model of chromosomal DNA packaging in the nucleus of a eukaryotic cell. (*a*) Main protein complexes involved in histone transportation and nucleosome formation. In the model, nucleosomes assemble on DNA from H2A ⋅ H2B and H3⋅ H4 histone dimers, which are transported by histone-binding chaperones schematically indicated as c_1_ and c_2_ in the figure. Specifically, by picking up newly synthesized histone dimers in the cell cytoplasm, chaperones switch from an unloaded state (c_1_ u or c_2_ u) to a histone-bound state (c_1_ b or c_2_ b, respectively). Then, by moving through NPCs, chaperones enter the cell nucleus, where they can deposit histone dimers onto the chromosomal DNA via nucleosome assembly. In addition to histone-binding chaperones, NPCs also allow free diffusion of ions and small metabolites between the cell nucleus and cytosol, helping to maintain the stability of the ionic microenvironment in the nucleoplasm. The bottom part of (*a*) shows the summary chemical equation of nucleosome assembly, which proceeds in several substeps detailed in (50, 51, 52, 53). (*b*) Conformations of chromosomes. In the model, conformations of chromosomes are described by triplets of unit vectors, $\left({\text{x}}^{\left(u\right)}\left(s_{u}\right)\text{, }{\text{y}}^{\left(u\right)}\left(s_{u}\right)\text{, }{\text{z}}^{\left(u\right)}\left(s_{u}\right)\right)$, u=1,…,Q, with each triplet indicating the orientation of the respective (u^th^) DNA molecule with reference to the fixed coordinate system, $\left({\text{x}}_{0}\text{, }{\text{y}}_{0}\text{, }{\text{z}}_{0}\right)$, at a point corresponding to the arc length $s_{u}\in \left[0\text{, }L_{u}\right]$ along the DNA. Here, $L_{u}$ is the length of the $u^{\text{th}}$ DNA molecule. (*c*) Discretized polymer model of DNA. To calculate the partition function, each DNA polymer was represented by a polygonal chain comprised of small straight segments, each of which was treated as a rigid body with an attached local Cartesian coordinate frame, $\left({\text{x}}_{j}\text{, }{\text{y}}_{j}\text{, }{\text{z}}_{j}\right)$, representing the DNA segment orientation in space with respect to the fixed global coordinate system, $\left({\text{x}}_{0}\text{, }{\text{y}}_{0}\text{, }{\text{z}}_{0}\right)$.

Packaging of long chromosomal DNA into a tiny nuclear space in eukaryotic cells is mainly done with the help of special DNA architectural proteins, histones. The process of nucleosome assembly, which is schematically shown in Fig. 1 *a*, usually proceeds in several substeps (50, 51, 52, 53), relying on the help of histone-binding chaperones, such as Asf1 or Nap1, involved in transportation of H2A ⋅ H2B and H3 ⋅ H4 histone dimers (54,55). Correspondingly, in the model we considered two types of chaperones: 1) c_1_ chaperones that can either be in empty/unloaded state (c_1_ u) or histone-bound state (c_1_ b), in which they form a complex with an H2A⋅ H2B dimer, and 2) c_2_ chaperones that also can be either in unloaded state (c_2_ u) or histone-bound state (c_2_ b), but this time forming a complex with an H3⋅ H4 dimer (see schematic in Fig. 1 *a*). Since experimental data suggest that some histone-binding chaperones, such as Nap1, shuttle between the nucleus and cytosol in eukaryotic cells (56), in our model it was assumed for simplicity that both histone-loaded and unloaded chaperones c_1_ and c_2_ can move through nuclear pore complexes (NPCs) without any restriction, see Online Appendix M for more details.

Since nuclei of living cells grown in a three-dimensional (3D) extracellular matrix that mimics the natural microenvironment take on almost spherical shapes (25,57), the cell nucleus in the model was represented by a spherically shaped NE enclosing nucleoplasm of $V_{\text{nucl}}$ volume. The nucleus itself was submerged into the cell cytosol of $V_{\text{cyto}}$ volume, which served as a buffer cushioning changes in the nucleoplasmic concentrations of unloaded and histone-bound chaperones that may result from rearrangement of chromatin structure.

Besides chaperones, the cytosol also mitigated changes in the nucleoplasmic levels of Na^+^, K^+^, and Cl^−^ ions, which have a major effect on the strength of DNA-histone interactions and chromatin organization (43,44,46). Microelectrode measurements, as well as radioautographic and extractive analysis, suggest that these monovalent ions can rather freely move between the cell nucleus and cytosol (58,59). Furthermore, their cytosolic concentrations are usually kept at nearly constant levels by transmembrane ion pumps and ion channels to maintain electroneutrality of intracellular environment and at the same time to counterbalance osmotic pressure created by the cell metabolites and proteins onto the cell membrane (7,8,60). Following previously published experimental and theoretical studies (6,60,61), the total cytosolic concentrations of monovalent Na^+^ and K^+^ ions on one hand, and Cl^−^ ions and negatively charged cell metabolites on the other hand, were both put equal to $c_{\text{ions}}=150$ mM in all our calculations.

As for the chromosomal DNA, it was modeled as a set of polymers constrained inside the cell nucleus, whose total number (*Q*) matches the number of chromosomes in the studied eukaryotic cells (in human cells Q=46), with the total length of DNA pieces ($L=\sum_{u=1}^{Q}L_{u}$) being equal to the total size of the cell genome. Here, $L_{u}$ is the length of the $u^{\text{th}}$ DNA polymer comprising the $u^{\text{th}}$ chromosome. Thus, to describe chromatin organization in human cells the total length of all DNA polymers was set equal to L=6.2 Gbp in all calculations (62).

3D conformations of the DNA polymers were represented in the model by $R^{\left(u\right)}\left(s_{u}\right)$, u=1,…,Q, functions, where $s_{u}\in \left[0\text{,}\;L_{u}\right]$ is the arc length along the $u^{\text{th}}$ DNA polymer and $R^{\left(u\right)}$ is a 3D Euler rotation matrix assigned to each point residing on the contour of the $u^{\text{th}}$ chromosome, which is a function of the arc length, $s_{u}$. This matrix indicates orientation of the DNA backbone at the corresponding point with respect to the global coordinate system, $\left({\text{x}}_{0}\text{,}\;{\text{y}}_{0}\text{, }{\text{z}}_{0}\right)$, such that the unit vector ${\text{z}}^{\left(u\right)}\left(s_{u}\right)=R^{\left(u\right)}\left(s_{u}\right)\;{\text{z}}_{0}$ resulting from the rotation of ${\text{z}}_{0}$ axis of the global coordinate system via Euler matrix $R^{\left(u\right)}\left(s_{u}\right)$ is tangential to the DNA backbone; whereas the other two unit vectors, ${\text{x}}^{\left(u\right)}\left(s_{u}\right)=R^{\left(u\right)}\left(s_{u}\right)\;{\text{x}}_{0}$ and ${\text{y}}^{\left(u\right)}\left(s_{u}\right)=R^{\left(u\right)}\left(s_{u}\right)\;{\text{y}}_{0}$, are normal to the DNA backbone, keeping track of the DNA twist angle (see Fig. 1 *b*).

It should be noted that, as in our previous work (49), the main results of this study do not depend on the precise details of the multimeric structures of protein complexes, such as histone octamers. For this reason, nucleosomes formed on the chromosomal DNA were represented in the model by solenoid-like structures schematically shown in Fig. 1 *b*, whose geometry match that of DNA wrapped around histone octamers in nucleosome complexes (63,64).

We then used a statistical physics approach to calculate the partition function of the system to describe the conformation of chromatin. Indeed, assembly of nucleoprotein complexes on DNA is mainly governed by DNA-binding affinities and positioning entropies of proteins (50, 51, 52, 53,65). Moreover, it has been shown previously that methods of statistical physics seem to correctly predict positions of nucleosomes on the chromosomal DNA near transcription start sites as well as on the global genomic scale (66, 67, 68, 69, 70). This observation is further supported by numerous theoretical studies of a large-scale chromatin organization based on thermodynamic equilibrium calculations, which demonstrated good agreement with the existing experimental data (see, for example (30,31,33,35,37, 38, 39,41,71,72)). Finally, theoretical estimations based on previously reported experimental measurements indicate that, under physiological conditions, active nuclear transportation processes should have a negligible effect on the near-equilibrium nucleocytoplasmic distributions of mobile histone-binding chaperones, see Online Appendix M. Altogether, these results suggest that the statistical physics/equilibrium thermodynamics approach provides a physically accurate description of the chromosomal DNA organization in nuclei of living cells, which for this reason was adopted in our study.

To calculate the partition function, *Z*, we utilized an implicit solvent method, which leads to the following formula (see (73) and Online Appendix A for more details):

(1) $$\begin{array}{c} Z=\sum_{\text{DNA}-\text{protein}}\prod_{j}\left[\frac{1}{n_{j}!}\prod_{k=1}^{n_{j}}\int_{V_{j}}\frac{Z_{j}^{\text{in}}\text{d}r_{jk}}{{\text{Λ}}_{j}^{3}}\right]\\ \times \prod_{u=1}^{Q}\left[\int_{V_{\text{nucl}}}\text{d}r_{\text{0}}^{\left(u\right)}\int DR^{\left(u\right)}\;e^{-\beta E_{u}[R^{\left(u\right)}]}\right]\\ \times e^{-\frac{\beta }{2}\int_{R^{3}}\text{d}r\int_{R^{3}}\text{d}r^{\text{′}}\;\rho_{\text{e}}\left(r\right)U_{\text{e}}\left(r-r^{\text{′}}\right)\rho_{\text{e}}\left(r^{\text{′}}\right)} \end{array}.$$

Here, $\Sigma_{\text{DNA−protein}}$ is the sum over all possible ways, in which nucleosomes can be positioned on chromosomal DNA. $\underset{j}{\prod }$ is the product over all types of particles (ions, proteins, cell metabolites, etc.) diffusing inside the cell, which are enumerated by index *j*. $n_{j}$ is the total number of particles of type *j* inside the cell. $\prod_{k=1}^{n_{j}}$ is the product over all particles of the same type, *j*, whose positions in space are described by the radius vectors $r_{jk}$. Integration $\underset{V_{j}}{\int }{\text{d}r}_{jk}$ in the above formula is performed over the volume $V_{j}$ accessible to particles of type *j*. In the case of ions, small proteins, and cell metabolites, $V_{j}$ corresponds to the union of the cell nucleus and cytosol volumes: $V_{j}=V_{\text{nucl}}\cup \;V_{\text{cyto}}$; whereas, in the case of macromolecular complexes that cannot move through NPCs into the nucleoplasm, we have $V_{j}=V_{\text{cyto}}$. ${\text{Λ}}_{j}$ and $Z_{j}^{\text{in}}$ are thermal de Broglie wavelength and the partition function describing inner degrees of freedom of the $j^{\text{th}}$ type of particles, respectively. $\prod_{u=1}^{Q}$ is the product over all chromosomes confined inside the nucleus, whose 3D conformations are represented by ${\text{R}}^{\left(u\right)}$ functions. ${\text{r}}_{0}^{\left(u\right)}$ is the radius vector describing the position of the starting end of the DNA polymer comprising the $u^{\text{th}}$ chromosome. $\int DR^{\left(u\right)}$ are Feynman-like path integrals along the contours of the respective DNA polymers (u=1,…,Q), which are calculated over all possible chromosome conformations. $E_{u}\left[R^{\left(u\right)}\right]$ is the energy of the DNA polymer comprising the $u^{\text{th}}$ chromosome, which incorporates elastic deformation of the DNA as well as its interaction with DNA-binding proteins (histones) (see Online Appendices A and B). In the general case, $E_{u}$ energy is determined by the 3D conformation of the $u^{\text{th}}$ chromosome, which is described by $R^{\left(u\right)}\left(s_{u}\right)$ function. $\beta =1/k_{\text{B}}T$ is the inverse thermodynamic temperature, where $k_{\text{B}}$ is the Boltzmann constant and *T* is temperature of the surrounding environment. $U_{\text{e}}\left(r\right)=1/4\pi \varepsilon r$ is the core part of the electrostatic potential, where r=‖r‖ is the length of the distance vector, r, between the electrically charged particles. *ε* is permittivity of the intracellular medium, which in the model was put equal to water permittivity. Finally, $\rho_{\text{e}}\left(r\right)$ is the distribution of electrical charges inside the cell, which is described by Eq. A4 (Online Appendix A). More details regarding the partition function as well as treatment of the volume-exclusion effect can be found in Online Appendices A and L.

To calculate the partition function given by Eq. 1, in this study we employed the mean field approach by considering randomly fluctuating electrostatic field, *ψ*, created by electrically charged ions, metabolites, and DNA, based on which it can be shown that Eq. 1 can be reduced to the following formula (see Online Appendix A):

(2) $$\text{ln}\;Z=\text{ln}\;Z_{\psi_{\text{sp}}}-\beta \sum_{j}n_{j}\mu_{j}^{\psi_{\text{sp}}},$$

where $Z_{\psi_{\text{sp}}}$ and $\mu_{j}^{\psi_{\text{sp}}}$ are the partition function of DNA and the electrochemical potential of the $j^{\text{th}}$ type of particles in the presence of the stationary phase electrostatic field, $\psi_{\text{sp}}$, which solves the following functional equation (see Online Appendix A):

(3) $$\frac{1}{4\pi \beta r^{2}}\frac{\delta \text{ln}Z_{\psi }}{\delta \psi \left(r\right)}+2q_{\text{e}}c_{\text{ions}}\text{sinh}\left[\beta q_{\text{e}}\psi \left(r\right)\right]-\varepsilon \Delta \psi \left(r\right)=0.$$

Here, *r* is the radial distance measured from the center of the cell nucleus. $q_{\text{e}}=1.6\times 10^{-19}$ C is the elementary charge. $Z_{\psi }$ is the partition function of DNA in the presence of an electrostatic field, *ψ* (Online Appendices B–D). $\frac{\delta \text{ln}Z_{\psi }}{\delta \psi \left(r\right)}$ is the functional derivative of the DNA partition function, $Z_{\psi }$, with respect to the electrostatic field, *ψ* (Online Appendix E).

To solve Eq. 3, the electrostatic field ψ(r) was represented in our study in a form of the following Fourier-Bessel expansion series:

(4) $$\psi \left(r\right)=\frac{\psi_{0}}{2}\left[1-\text{tanh}\left(\frac{r-R_{0}}{w}\right)\right]+\sum_{n=1}^{n_{\text{max}}}\psi_{n}j_{0}\left(\frac{\pi nr}{R_{\text{nucl}}}\right),$$

where $R_{\text{nucl}}$ is the radius of the cell nucleus. $n_{\text{max}}$ is the total number of Fourier-Bessel modes used in calculations (in this study, $n_{\text{max}}=18$). As for $R_{0},w,\psi_{0},\psi_{1},\ldots ,\psi_{n_{\text{max}}}$, these are the model fitting parameters, whose values were determined by Nelder-Mead simplex algorithm (74), which was used to minimize deviation of the left side of Eq. 3 from zero, see Online Appendix A.

Finally, to calculate the partition function of chromosomal DNA, $Z_{\psi }$, in an arbitrary electrostatic potential field, *ψ*, we used a discretized semiflexible polymer chain model in which DNA is partitioned into short straight segments, whose size (b=3.4 nm) is much smaller than the bending persistence length of DNA (A=50 nm (75,76)) (see schematic in Fig. 1 *c*). Since the elastic bending and twisting energies of DNA can be described in terms of relative orientations of adjacent DNA segments, it is then possible to utilize transfer-matrix calculations to obtain the partition function of DNA, and it can be shown that, up to a non-essential prefactor (see Online Appendices B–E):

(5) $$Z_{\psi }\propto \prod_{u=1}^{Q}\left[UL^{N_{u}-1}Y\right],$$

where $N_{u}$ is the total number of segments in a discretized polygonal chain representing the DNA polymer comprising the $u^{\text{th}}$ chromosome. **U** and **Y** are boundary condition vectors that depict physical states of the DNA end segments in each chromosome. **L** is a transfer-matrix, which characterizes DNA segments’ interactions with DNA-binding proteins and the surrounding electrostatic potential field, *ψ*, as well as describing local bending and twisting elasticities of DNA.

The main advantage of Eq. 5 is that the value of each matrix product $UL^{N_{u}-1}Y$ is mainly determined by the dominant eigenvalue, $\lambda_{\text{max}}$, of the transfer-matrix **L** when $N_{u}\gg 1$, which is the case for long DNA molecules found in eukaryotic cells. Specifically, it can be shown that $Z_{\psi }=C_{0}\lambda_{\text{max}}^{N_{\text{tot}}}$, where $N_{\text{tot}}=\sum_{u=1}^{Q}N_{u}$ is the total number of DNA segments in all of the chromosomes, and $C_{0}$ is a constant independent of $N_{\text{tot}}$. Since both $C_{0}$ and the dominant eigenvalue, $\lambda_{\text{max}}$, can be found with the help of standard techniques, such as the power iteration method (77) (also see Online Appendices E and I), this makes it possible to calculate the partition function of an arbitrarily long DNA in the presence of DNA-binding proteins and electrostatic interactions between all of the system components—a task that could not be handled by any of the theoretical methods previously employed in studies of the large-scale chromatin organization in nuclei of living cells. Furthermore, the above approach provides direct connection between the mesoscale structure of the entire cell genome and properties of individual protein complexes, which could not be achieved in previous studies.

In addition, it should be noted that, in contrast to previous theoretical works (30, 31, 32, 33, 34, 35, 36, 37, 38, 39, 40, 41,71,72), in our model the chromatin structure is not fixed and nucleosomes are allowed to form/disassemble anywhere on DNA, enabling evaluation of the grand partition function of DNA over all possible DNA-protein conformations, which so far has been considered a problem that cannot be solved for genome-size DNA by using modern computational systems.

By taking derivatives of the logarithm of the partition function defined by Eq. 2 with respect to various model parameters, such as the volume of the cell nucleus or the histones’ binding energy to DNA, etc., it is possible to find the mean pressure created by DNA on the NE, the average DNA conformation and occupancy by nucleosomes, and many other quantities characterizing the physical states of the chromosomal DNA and the cell nucleus. For example, the average pressures of the chromosomal DNA, monovalent ions, and cytosolic macromolecules on the NE, $p_{\text{DNA}}$, $p_{\text{ions}}$, and $p_{\text{macro}}$, can be found via the following formulas (Online Appendix K):

(6) pDNA=−∂GDNA∂VnuclT=const∀j:nj=constpions=−∂Gions∂VnuclT=const∀j:nj=constpmacro=−∂Gmacro∂VnuclT=const∀j:nj=const,

where $G_{\text{DNA}}$, $G_{\text{ions}}$, and $G_{\text{macro}}$ are the free energies of DNA, monovalent ions, and cytosolic macromolecules, respectively (Online Appendix K):

(7) $$G_{\text{DNA}}=-k_{\text{B}}T\text{ln}Z_{\psi_{\text{sp}}}\;\;G_{\text{ions}}=\sum_{\text{monovalent ions}}n_{j}\mu_{j}^{\psi_{\text{sp}}}\;G_{\text{macro}}=\sum_{\text{macro}-\text{molecules}}n_{j}\mu_{j}^{\psi_{\text{sp}}}.$$

In addition, if a small part of DNA is stretched by force *F*, it is possible to calculate the force-dependent partition function of DNA ($Z_{\psi_{\text{sp}}F}$) by utilizing a modified version of Eq. 5 (i.e., Eq. I6 in Online Appendix I), and as a result obtain the end-to-end extension of the stretched DNA part (*z*) as well as its occupancy fraction by nucleosomes ($O_{\text{nucl}}$) with the help of the following formulas:

(8) $$z\left(F\right)=-\frac{\partial G_{\text{DNA}}}{\partial F}=k_{\text{B}}T\frac{\partial \text{ln}Z_{\psi_{\text{sp}},F}}{\partial F}\;O_{\text{nucl}}=-\frac{K}{N}\;\frac{\partial G_{\text{DNA}}}{\partial \mu_{\text{pr}}}=\frac{K}{\beta N}\;\frac{\partial \text{ln}Z_{\psi_{\text{sp}},F}}{\partial \mu_{\text{pr}}}.$$

Here, *K* is the number of DNA segments constrained in a single nucleosome, and *N* is the total number of DNA segments in the studied DNA molecule. $\mu_{\text{pr}}$ is the binding free energy of histone octamers to DNA.

In a very similar way, the mean-square displacement (MSD) between any two points residing on the DNA contour can be calculated as:

(9) $$\text{MSD}=\left\langle \Delta x^{2}+\Delta y^{2}+\Delta z^{2}\right\rangle =-\frac{3}{\beta }\;{\frac{\partial^{2}G_{\text{DNA}}}{\partial F^{2}}|}_{F=0}=\frac{3}{\beta^{2}}{\frac{\partial^{2}\text{ln}Z_{\psi_{\text{sp}}}}{\partial F^{2}}|}_{F=0}.$$

More details regarding the above formulas can be found at the end of Online Appendix I. The values of the model parameters used in calculations are shown in Table SI.

The source code of the computer programs can be downloaded from http://www.artem-efremov.org.

Finally, it should be noted that, although here we have mainly focused on description of the model approach to study DNA organization in nuclei of living cells, the same method, with minor modifications, can be also employed to gain insights into DNA packaging in viral particles (see the following section and Online Appendix A).

## Results

### Viral DNA packaging

**Figure 2:**
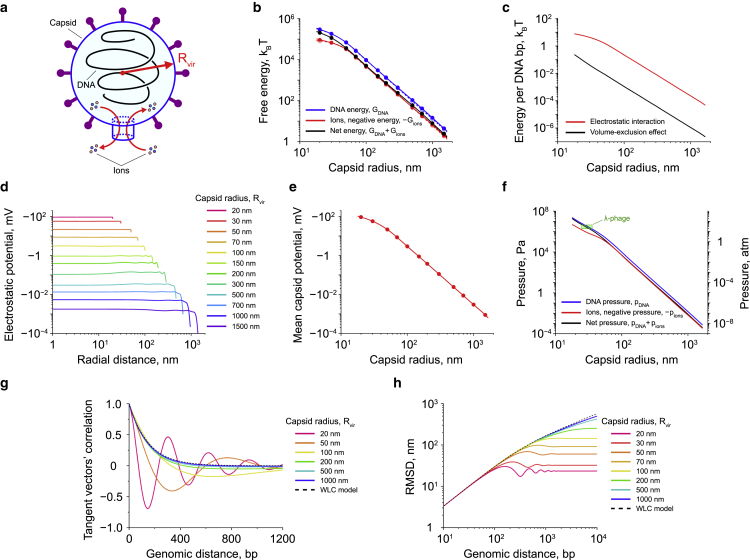
DNA packaging in viral particles. (*a*) Schematic picture of DNA packaging in a viral particle. Due to the tight capsid space, DNA adopts a coil-like conformation (78, 79, 80, 81, 82). (*b*) Energy contributions made by DNA (*blue curve*) and ions (*red curve*) into the total free energy of a viral particle (*black curve*) as functions of the capsid size. Data points shown in the graph are the results of transfer-matrix calculations; whereas lines indicate smoothing spline interpolation. Solid curves correspond to the case of a viral DNA that has the bending and twisting persistence lengths corresponding to those of B-DNA: A=50 nm (75,76) and C=95 nm (86, 87, 88), respectively; whereas dashed curves demonstrate results obtained for a freely moving joint polymer chain of the same length but with zero bending and twisting elasticities between the polymer segments. All energy values are shown in the graph up to a nonessential additive constant. (*c*) Electrostatic and volume-exclusion DNA interaction energies per single DNA basepair as functions of the capsid size. The curves are calculated using Eq. L28 from Online Appendix L. (*d* and *e*) Spatial distribution and volume-averaged value of the electrostatic potential in viral particles of different sizes. Each data point shown in (*e*) corresponds to a curve from (*d*). The solid line in (*e*) demonstrates the smoothing spline interpolation of the data points. (*f*) Pressure generated by DNA and ions on the walls of a viral particle at different capsid sizes. The curves are calculated based on the data shown in (*b*). The green rectangle indicates the area corresponding to the experimentally measured range in *λ*-phage particles (83,84). (*g* and *h*) The average correlation function between a pair of unit vectors tangent to the DNA backbone and the root mean-square distance (RMSD) between two points located on the DNA as functions of the genomic distance along the DNA. In all computations, the DNA length was 14 *μ*m, which corresponds to the size of EMBL3 *λ*-phage genome of 41.5 kbp (83). The size of DNA segments in the discretized polymer model was b=3.4 nm.

To test the theoretical framework developed in this study, we first applied it to describe a simpler case of DNA packaging in viral particles, comparing the calculation results with the following known facts: 1) the viral DNA inside viral particles is folded into a coil-like structure (78, 79, 80, 81, 82) (see also Fig. 2 *a*); 2) the amount of pressure applied by the folded viral DNA to the capsid wall is of the order of ∼10−40 atm (83,84), and 3) this pressure is mainly generated by the DNA-DNA electrostatic repulsion, which makes a dominant contribution to the total energy of viral particles (85).

To this aim, we first checked the relative contribution of the elastic deformation energy of DNA into the total energy of a viral particle. By varying the bending and twisting elasticities of viral DNA from the values corresponding to B-DNA to zero, it has been found that elastic deformations of the folded viral DNA make a negligible contribution to the total free energy of a viral particle at all particle sizes in the range of 20−1000 nm (see Fig. 2 *b*).

Further calculations have shown that the vast majority of the free energy of a viral particle comes from electrostatic interactions between the system components, such as DNA and diffusing ions, which are by ∼1 to 2 orders of magnitude stronger than the DNA-DNA volume-exclusion effect (Fig. 2 *c*). This result is in good agreement with previous studies (85) and seems to be caused by a rapid increase in the electrostatic potential of viral particles as their size decreases (Fig. 2, *d* and *e*).

Next, we evaluated the pressure exerted by DNA and ions on the capsid wall. As can be seen from Fig. 2 *f*, the pressure curve calculated for viral DNA passes exactly through the center of the experimentally measured range in *λ*-phages, suggesting that our model provides an accurate description of the physical properties of the packed DNA.

Finally, we estimated the correlation between unit vectors tangent to the DNA backbone as well as the root mean-square distance (RMSD) between two points residing on DNA as functions of the genomic distance along the DNA (Fig. 2, *g* and *h*). It has been found that, with a decrease in the size of the viral capsid, the DNA conformation becomes more and more ordered; and, starting from $R_{\text{vir}}\approx 50$ nm, the correlation function between tangent vectors takes the form of damped oscillations (Fig. 2 *g*), which indicates DNA folding into a coil-like conformation. The RMSD plot shown in Fig. 2 *h* further confirms this observation, suggesting that our model provides a physically meaningful description of DNA packaging in viral particles, which is in good agreement with known experimental data (80,82).

Interestingly, the RMSD graphs displayed in Fig. 2 *h* look very similar to those reported in a recent study of polymer molecules confined in 2D spaces (89), indicating strong similarity between polymers’ behavior in 2D and 3D cases.

Altogether, the above results demonstrate that the developed model correctly describes behavior of DNA confined in a tight space, making it suitable for study of DNA packaging in viral particles and nuclei of living cells. We then used the developed theoretical framework to gain understanding of molecular mechanisms responsible for regulation of the cell nucleus size and its potential downstream effects on chromatin structure.

### Mechanical equilibrium of the cell nucleus

**Figure 3:**
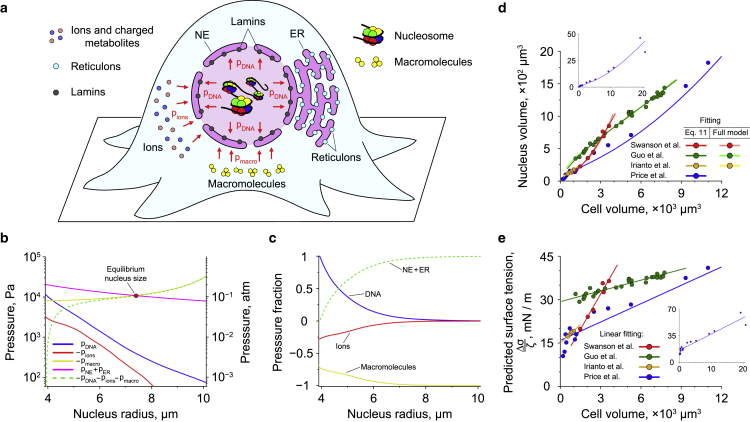
Cell nucleus size regulation. (*a*) Schematic picture of cellular components exerting pressure on the NE. In general, the nucleus size is determined by mechanical equilibrium between the pressures created by DNA ($p_{\text{DNA}}$), ions ($p_{\text{ions}}$) and macromolecules ($p_{\text{macro}}$). In addition, it has been previously suggested that the NE and ER may be involved in a tug-of-war with each other over the shared lipid membrane (1), which can be described in terms of $p_{\text{NE}}$ and $p_{\text{ER}}$ pressures associated with the surface tensions of the NE and ER membrane. (*b*) Pressures exerted on the NE by DNA, ions, macromolecules, and the surface tensions of the NE and ER membrane as functions of the nucleus size. The yellow curve, which represents the osmotic pressure created by cytosolic macromolecules, is plotted for the case of $n_{\text{macro}}=10^{10}$ molecules. The magenta curve demonstrates the net pressure, $p_{\text{NE}}+p_{\text{ER}}$, generated by Δσ=20 mN/m difference in the surface tensions of the NE and ER membrane; whereas the green dashed curve indicates the equilibrium value of the net pressure, $p_{\text{NE}}+p_{\text{ER}}=-\;p_{\text{DNA}}-p_{\text{ions}}-p_{\text{macro}}$ (Eq. 10). In the calculations, the total length of DNA was set equal to 2.1 m, which corresponds to the size of the human genome of ∼6.2 Gbp (62). The size of DNA segments in the discretized polymer model was b=3.4 nm. (*c*) Relative contributions of different cell components to the total positive/negative pressure acting on the NE calculated based on the data shown in (*b*). Results displayed in (*a*) and (*b*) were obtained in the case of a cell with the total volume of $V_{\text{cell}}=8000$*μ*m^3^. Smaller cells ($V_{\text{cell}}=4000$*μ*m^3^) demonstrate very similar behavior (see Fig. S1). (*d*) Correlation between the nucleus and cell volumes. Solid curves indicate fitting of experimental data from (19,20,22,25) either to the full model described by Eq. 10 or to the reduced model described by Eq. 11. In the former case, the fitting was done by varying a single model parameter—the difference between the surface tensions of the NE and ER membrane, Δσ; whereas, in the latter case, the linear fitting of the Δσ/ζ ratio shown in (*e*) was used as an input to Eq. 12. The inset displays a large-scale view of the fitting of the experimental data from (19). (*e*) Δσ/ζ ratio as a function of the nucleus size. Data points show the values calculated using Eq. 11 based on experimental data presented in (*d*). Solid lines indicate linear fitting of the data points. The inset demonstrates a large-scale view of the fitting of the data points obtained based on (19).

In the general case, the nucleus size is determined by mechanical equilibrium corresponding to a net zero pressure acting on the NE. This includes the pressure created by chromosomal DNA ($p_{\text{DNA}}$) (10,12), as well as the osmotic pressure resulting from the gradients of electrically charged ions and metabolites across the NE ($p_{\text{ions}}$), which develop due to the negative electrostatic potential of the cell nucleus with respect to the cytoplasm (59,90,91) (see Fig. 3 *a*). Cytosolic macromolecules that cannot freely move through NPCs into the nucleus have also been shown to exert outside osmotic pressure on the NE ($p_{\text{macro}}$) (9,11). In addition, as mentioned in the introduction, it has been previously hypothesized that ER-binding proteins, such as reticulons, and the lamina network that inlays the NE, may be involved in a tug-of-war over the lipid membrane shared by the NE and ER in metazoan cells (1). From a physical point of view, this tug-of-war can be described in terms of opposite pressures, $p_{\text{NE}}=-4\sigma_{\text{NE}}/R_{\text{nucl}}$ and $p_{\text{ER}}=4\sigma_{\text{ER}}/R_{\text{nucl}}$, acting on the NE, which are created by the surface tensions of the NE and ER membrane, $\sigma_{\text{NE}}$ and $\sigma_{\text{ER}}$, see Online Appendix K.

Summing all the above pressure terms, it is easy to obtain the following formula for mechanical equilibrium of the NE:

(10) $$p_{\text{DNA}}+p_{\text{ions}}+p_{\text{macro}}+p_{\text{NE}}+p_{\text{ER}}=0.$$

By using Eqs. 6 and 10, we then plotted the above pressures as functions of the nucleus radius, $R_{\text{nucl}}$ (see Figs. 3 *b* and S1 *a*). From the graph it can be seen that the equilibrium size of the nucleus is predominantly determined by the difference in the surface tensions of the NE and ER, as well as the osmotic pressure generated by cytosolic macromolecules. Indeed, from direct comparison of relative contributions of various cell components into the total positive and negative pressures acting on the NE (Figs. 3 *c* and S1 *b*) it can be concluded that neither DNA nor ions make a substantial contribution to the regulation of the nucleus size in a wide range of model parameters. Only in the case when the nucleus is compressed strong enough, does the model predict that DNA starts to produce sufficiently large repulsing force counteracting the applied pressure. This result is in good agreement with previous experimental studies, which showed that, in many living cells, DNA does not affect the nucleus size, rather setting its minimum possible value (9,21,27, 28, 29).

Hence, in a wide range of experimental conditions, an approximate equilibrium radius of the nucleus, $R_{\text{nucl}}$, can be found as a unique real-valued solution of the following cubic equation that can be derived from Eq. 10:

(11) $$p_{\text{NE}}+p_{\text{ER}}+p_{\text{macro}}=0\;\Rightarrow \;\frac{4\Delta \sigma }{R_{\text{nucl}}}=\frac{n_{\text{macro}}k_{\text{B}}T}{V_{\text{cell}}^{\text{osm}}-\frac{4\pi }{3}R_{\text{nucl}}^{3}},$$

and thus

(12) $$R_{\text{nucl}}=\sqrt[{3}]{-\frac{q}{2}+\sqrt{\frac{q^{2}}{4}+\frac{p^{3}}{27}}}-\sqrt[{3}]{\frac{q}{2}+\sqrt{\frac{q^{2}}{4}+\frac{p^{3}}{27}}},$$

where $\text{Δ}\sigma =\sigma_{\text{ER}}-\sigma_{\text{NE}}$ is the difference in the surface tensions of the NE and ER membrane. $V_{\text{cell}}^{\text{osm}}=V_{\text{nucl}}+V_{\text{cyto}}$ is the osmotically active volume of a cell, which typically occupies ∼70% of the total cell volume ($V_{\text{cell}}$): $V_{\text{cell}}^{\text{osm}}\approx 70\text{\%}\cdot V_{\text{cell}}$ (22,92). $n_{\text{macro}}$ is the total number of macromolecules in the cell cytosol. Taking into account that most of the macromolecules are either sufficiently large proteins or protein complexes that cannot move through NPCs, it is natural to expect that their number is approximately proportional to the total number of proteins in a living cell: $n_{\text{macro}}\approx \zeta n_{\text{pr}}=\zeta c_{\text{pr}}V_{\text{cell}}$. Here, *ζ* is a proportionality coefficient (0<ζ<1), and $c_{\text{pr}}$ is the average protein concentration in living cells: $c_{\text{pr}}=2.7\times 10^{6}$ proteins/*μ*m^3^ (93). Finally, *q* and *p* coefficients in Eq. 12 are: $q=-3V_{\text{cell}}^{\text{osm}}/4\pi$ and $p=3\zeta c_{\text{pr}}V_{\text{cell}}k_{\text{B}}T/\pi \text{Δ}\sigma$.

We then used Eq. 11 to estimate the Δσ/ζ ratio based on experimentally measured volumes of different types of cells and their nuclei (19,20,22). The calculation results are shown in Fig. 3 *e*, from which it can be seen that the Δσ/ζ ratio predicted by the model changes almost linearly with the cell volume in all considered cases. As a result, by fitting each of the data set shown in Fig. 3 *e* to a linear function and then substituting this function into Eq. 12, a nearly perfect fit of the experimentally measured correlations between the cell and nucleus volumes could be achieved (see Fig. 3 *d*). Fitting of the experimental data to the full model described by Eqs. 6 and 10 led to very similar results (Fig. 3 *d*), indicating the robustness of Eqs. 11 and 12.

Interestingly, the obtained values of Δσ/ζ turned out to be ∼20−40 mN/m (Fig. 3 *e*), which is close to the elastic modulus of the NE measured in isolated nuclei of *Xenopus laevis* oocytes (28±8 mN/m (9)). This result suggests that the nuclear lamina network likely plays a major role in governing the nucleus size regulation in *Xenopus* oocytes—a model prediction—which is in good agreement with previous experimental studies (14,15).

Using Eq. 10, we also estimated Δσ that has to be maintained by a living cell to retain the nucleus size at a specific value in the case of ζ=0.4−0.5 (see Fig. S1 *c*). From the figure it can be seen that bigger nuclei require a considerably larger Δσ, which corresponds to a high surface free energy density of the NE of the order of ∼1 to 10 $k_{\text{B}}T/{\text{nm}}^{2}$ (∼4−40 mN/m). Since numeric estimations suggest that the surface tension of ER is likely to be small ($|\sigma_{\text{ER}}|<0.1$ mN/m (94)), large positive values of $\text{Δ}\sigma =\sigma_{\text{ER}}-\sigma_{\text{NE}}$ imply a high negative membrane tension of the NE. That is, the model predicts that the NE is strongly compressed in living cells due to the osmotic pressure created by cytosolic macromolecules rather than stretched as in membrane rupture experiments (95,96). As a result, it can be concluded that most of the external pressure exerted by cytosolic macromolecules on the NE will likely fall on the inlaying lamina network, which emphasizes the importance of the latter as a power source that drives the growth of the nucleus both throughout the cell cycle (14,15) and during differentiation of embryonic stem cells (97).

### Effect of the nucleus size on chromatin

**Figure 4:**
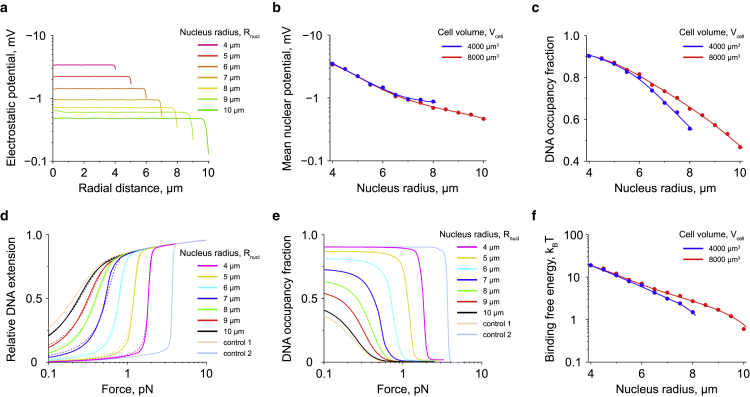
Nuclear electrostatic potential and nucleosome stability as functions of the nucleus size. (*a*) Distribution of the electrostatic potential in nuclei of different sizes. The plot shows results obtained for a cell with the total volume of $V_{\text{cell}}=8000$*μ*m^3^. In smaller cells ($V_{\text{cell}}=4000$*μ*m^3^), the nuclear electrostatic potential exhibits a similar distribution (see Fig. S2). (*b*) Volume-averaged value of the electrostatic potential in nuclei of different sizes. Data points indicate values calculated based on the curves shown in (*a*) and Fig. S2; whereas solid lines demonstrate smoothing spline interpolation. (*c*) Occupancy fraction of chromosomal DNA by nucleosomes as a function of the nucleus size. Data points represent results of transfer-matrix calculations; whereas solid lines show smoothing spline interpolation. (*d* and *e*) Force-extension and force-DNA occupancy fraction curves obtained by mechanical stretching of a small fragment of chromosomal DNA in nuclei of different sizes. Solid curves in (*d*) and (*e*), except for control curves 1 and 2, demonstrate results of transfer-matrix calculations performed for a cell with the total volume of $V_{\text{cell}}=8000$*μ*m^3^ (see also Fig. S3 for the $V_{\text{cell}}=4000$*μ*m^3^ case). Dashed curves in (*d*) indicate force-extension curves calculated for an isolated DNA fragment at different values of the binding free energy of histone octamers to DNA in the absence of the nuclear electrostatic potential (49). Control curve 1 shows the result of transfer-matrix calculations in the absence of electrostatic interactions for an infinitely large cell nucleus; whereas control curve 2 depicts transfer-matrix calculations for in vitro experiments performed in ATP-depleted and diluted *Xenopus* egg extract (49,98). (*f*) Binding free energy of histone octamers to DNA as a function of the cell nucleus size. The data points were calculated based on the force-extension curves from (*d*) and Fig. S3*a*, as specified in the main text. Solid curves indicate prediction of the binding free energy of histone octamers by Eq. 13.

Since existing experimental studies suggest that electrostatic interactions play a major role in DNA organization, we next checked how the size of the cell nucleus affects its mean electrostatic potential with respect to the cytoplasm. From the results shown in Fig. 4, *a* and *b* it can be seen that the absolute magnitude of this potential experiences a rapid increase with the decreasing size of the nucleus, reaching the value of several millivolts, which is within the experimentally measured range of the nuclear potential in *Xenopus* oocytes, MDCK cells and isolated murine pronuclei (from approximately −10 to 0 mV (59,90,91)).

As histone dimers possess a strong electrical charge ($∼+37.2\;q_{\text{e}}$, where $q_{\text{e}}=1.6\times 10^{-19}$ C), it is clear that such a nuclear electrostatic potential may have a profound effect on the stability of nucleosomes formed on the chromosomal DNA. Indeed, the model calculations demonstrate that the DNA occupancy by nucleosomes can experience considerable variation in response to changes in the nucleus size (Fig. 4 *c*), suggesting strong dependence of the histone binding free energy to DNA on the nuclear electrostatic potential.

In fact, it is not hard to obtain the following approximate formula for the total binding free energy of histone octamers to DNA, $\mu_{\text{pr}}^{\text{tot}}$, which is equal to the difference between the electrochemical potentials of the initial and final states of the protein complexes contributing to nucleosome assembly (see Online Appendices A and B):

(13) $$\mu_{\text{pr}}^{\text{tot}}\approx \mu_{\text{pr}}^{0}-q_{\text{oct}}\left\langle \psi \right\rangle +2k_{\text{B}}T\text{ln}\theta_{{\text{c}}_{1}}+2k_{\text{B}}T\text{ln}\theta_{{\text{c}}_{2}}+2k_{\text{B}}T\;\sum_{i=1,2}\text{ln}\left(\frac{V_{\text{cell}}^{\text{osm}}+V_{\text{nucl}}^{\text{pr}}\left[e^{-\beta q_{{\text{c}}_{i}\text{u}}\left\langle \psi \right\rangle }-1\right]}{V_{\text{cell}}^{\text{osm}}+V_{\text{nucl}}^{\text{pr}}\left[e^{-\beta q_{{\text{c}}_{i}\text{b}}\left\langle \psi \right\rangle }-1\right]}\right).$$

Here, $\theta_{{\text{c}}_{i}}=n_{{\text{c}}_{i}\text{b}}^{0}/n_{{\text{c}}_{i}\text{u}}^{0}$ (i=1,2) are occupancy ratios of histone-binding chaperones, where $n_{{\text{c}}_{1}\text{b}}^{0}$, $n_{{\text{c}}_{2}\text{b}}^{0}$, $n_{{\text{c}}_{1}\text{u}}^{0}$, and $n_{{\text{c}}_{2}\text{u}}^{0}$ are the total average numbers of c_1_ and c_2_ chaperones in histone-bound and unloaded states, respectively. $q_{\text{oct}}$ is the electrical charge of a histone octamer. $q_{{\text{c}}_{i}\text{b}}$ and $q_{{\text{c}}_{i}\text{u}}$ are the net electrical charges of the $i^{\text{th}}$ chaperone, c_*i*_, in histone-bound and unloaded states, correspondingly. ⟨ψ⟩ is the mean value of the nuclear electrostatic potential obtained by averaging over the nucleus volume. $V_{\text{nucl}}^{\text{pr}}$ is the volume of the part of the nucleus accessible to proteins (Online Appendix M). Finally, $\mu_{\text{pr}}^{0}\approx 7$ $k_{\text{B}}T$ is the standard Gibbs free energy of nucleosome formation in the presence of histone-binding chaperones under the standard experimental conditions ($\theta_{{\text{c}}_{1}}=\theta_{{\text{c}}_{2}}=1$ and ψ=0), which can be evaluated based on the experimentally measured rates of histone association and dissociation from DNA (50,51).

To test Eq. 13 and quantify the effect of the nuclear electrostatic potential on nucleosomes, we evaluated the stability of the latter by calculating the force-extension curve of a small part of chromatin inside the cell nucleus. Indeed, as was shown in previous experimental and theoretical studies, the binding free energy of histone octamers to DNA can be evaluated based on measurements of the force-extension curve of chromatin (49,98,99).

To this aim, in our model, a virtual pulling force, *F*, was exerted to a 2-*μ*m fragment of chromosomal DNA, and the DNA extension was calculated using Eq. 8. From the results shown in Fig. 4 *d* (*solid curves*) it can be seen that, to induce nucleosome unfolding in smaller nuclei, a much higher force must be applied to the DNA compared with the case of larger nuclei, indicating stabilization of nucleosomes by the nuclear electrostatic potential.

By comparing the obtained force-extension curves with those predicted for an isolated DNA fragment at different values of the binding free energy of histone octamers to DNA (*dashed curves*, Fig. 4 *d*) (49), we estimated the value of $\mu_{\text{pr}}^{\text{tot}}$ for nuclei of different sizes, see data points in Fig. 4 *f*. By plotting the curve predicted Eq. 13, it then could be seen that this equation accurately predicts the behavior of the binding free energy of histone octamers to DNA as a function of the nucleus size.

Remarkably, Fig. 4 *f* shows that the nuclear electrostatic potential has a profound effect on the binding free energy of histone octamers to DNA, increasing the stability of nucleosomes by ∼1−20 $k_{\text{B}}T$ in a nucleus size-dependent manner. Although this change in the binding free energy of histone octamers to DNA appears to be smaller than the ∼40 $k_{\text{B}}T$ binding energy estimated based on in vitro single-DNA manipulation experiments (98), it can still reach values that are ∼1−3 times higher than the standard Gibbs free energy of nucleosome formation in the presence of histone-binding chaperones ($\mu_{\text{pr}}^{0}\approx 7$ $k_{\text{B}}T$) (50,51). Thus, it can be expected that the nucleus size-dependent change in the binding free energy of histone octamers to DNA can potentially have a significant effect on the stability of nucleosomes in living cells.

It is also interesting to note that, from Eq. 13, it follows that not only the electrical charges of histones play an important role in the stabilization of nucleosomes by the nuclear electrostatic potential but also the electrical charges of histone-binding chaperones. Indeed, simple model calculations based on Eq. 13 show that, depending on the electrical charge of histone-binding chaperones, a living cell can switch between two regimes: 1) nucleus size-sensing, in which the binding free energy of histone octamers to DNA depends on the nucleus size, and 2) nucleus-size insensitive, in which the binding free energy of histone octamers to DNA becomes nearly independent from the nucleus size, indicating the existence of a previously unknown potential regulatory pathway of nucleosome stability.

Altogether, the above results suggest that, contingent on physical properties of histone-binding chaperones, living cells may have very diverse response to changes in the nucleus size in terms of chromatin structure, reorganization of which can be induced by variation in the nuclear electrostatic potential.

## Discussion

In this study, we have developed a general theoretical framework aimed at description of DNA packaging in nuclei of living cells, which allowed us to address the question of nuclear organization by taking into account main physical forces contributing to it. Unlike previous theoretical models, which mainly focus on one or a few specific elements that affect chromatin organization and nucleus size regulation, our method takes into account many physicochemical factors, such as electrostatic interactions between DNA, ions and proteins, elastic properties of DNA, assembly/disassembly of nucleoprotein complexes and their 3D structures, etc., combining them into an integrated theoretical framework. The calculation results not only explain a wide range of experimental observations, but also predict the existence of a previously unknown link between the nucleus size and chromatin structure.

It has been found that the nucleus size in higher eukaryotes is predominantly determined by a tug-of-war between the osmotic pressure exerted by cytosolic macromolecules on the NE and the difference between the surface tensions of the NE and ER membrane. This finding is in good agreement with previous experimental studies showing that cytosolic macromolecules and lamins play the central role in the nucleus size regulation in metazoan cells (4,9,11,14,15,100,101).

Although there may exist other molecular mechanisms which might contribute to the nucleus size regulation, it is likely that the tug-of-war between cytosolic macromolecules, NE, and ER will remain one of the main factors involved in this process. For example, it is known that yeasts do not have lamin analogs, and thus their nuclei do not possess a lamina network. Because of that, these cells have to rely on alternative mechanisms to regulate volumes of their nuclei, for instance, through accumulation of nuclear proteins, which may be used by cells to build up internal osmotic pressure on the NE counterbalancing the outside pressure created by cytosolic macromolecules (5). In this case, the equilibrium nucleus size will still be described by the same Eqs. 10 and 11, with an additional term corresponding to the osmotic pressure created by a mobile fraction of macromolecules confined inside the cell nucleus (Eq. K10). It then can be shown that, in lower eukaryotes, which presumably have a low surface tension of the NE due to the lack of the lamina network, the nucleus volume changes in proportion to the cell volume (102,103). In contrast, nuclei of higher eukaryotes exhibit more complex behavior (Fig. 3 *d*), indicating an important role of the surface tension of the NE in regulation of the nucleus size.

In addition, it has been previously proposed that the actin cytoskeleton may also contribute to regulation of the nucleus size in mammalian cells via application of mechanical forces to the NE (18,104). However, experimental studies show that the actin network typically generates a rather small mechanical stress (∼20−1000 Pa (22,105)), which is 1 to 3 orders of magnitude less than the pressure developed by other cell components on the NE (Fig. 3 *b*). Furthermore, it has been found that disruption of the actin filaments by cytochalasin D does not influence osmolarity-induced change in the nucleus size (11). Thus, while the actin cytoskeleton may affect the nucleus shape, it is unlikely to cause considerable changes in the nucleus volume by exerting pressure on the NE, leading instead only to small fluctuations of the nucleus size of the order of tens to hundreds of nanometers (106,107).

However, we would like to emphasize that our study does not exclude the possibility of a significant role of the actin cytoskeleton in the regulation of the nucleus size by indirect means. For example, by driving the process of cell spreading, which is accompanied by a change in the cell volume (18,22), the actin cytoskeleton can affect the average concentration of cytosolic macromolecules and, hence, the osmotic pressure created by them on the NE. Ultimately, this leads to a change in the cell nucleus volume. Furthermore, experimental studies indicate that the actin cytoskeleton also has a profound effect on the nucleocytoplasmic distributions and intracellular levels of lamins, histone-modifying enzymes, and even some transcription cofactors (26,104,108, 109, 110, 111), thus indirectly affecting the nucleus volume by modulating the values of $p_{\text{NE}}$, $p_{\text{DNA}}$, and $p_{\text{macro}}$ pressure terms in Eq. 10.

As found in our study, by influencing the average density of negatively charged DNA, the nucleus size determines the mean electrostatic potential of the cell nucleus with respect to the cytoplasm, which has a profound effect on the stability of nucleosomes (Figs. 4, S2, and S3). Indeed, since histone octamers carry a large electrical charge ($∼+148\;q_{\text{e}}$, where $q_{\text{e}}$ is the electrical charge of a proton), even a small variation in the average nuclear electrostatic potential by 1 mV leads to ∼148 meV =5.5 $k_{\text{B}}T$ change in the electrostatic potential energy of each histone octamer located inside the cell nucleus. On the other hand, the free energy of mobile histones changes to a much lesser extent due to their electrostatic screening by histone-binding chaperones, as well as due to localization of a large part of these protein complexes in the cell cytosol (112), beyond the reach of the nuclear electrostatic potential. This leads to a high sensitivity of nucleosome assembly to small variations in the nuclear electrostatic potential as it is mainly governed by the free energy gap between DNA-associated histone octamers and mobile histones carried by chaperones (50, 51, 52, 53).

As for other DNA-binding proteins, whose electrical charges are typically an order of magnitude smaller than those of histone octamers, it is clear that the nuclear electrostatic potential should have a much weaker effect on formation of nucleoprotein complexes by these proteins. Namely, 1 mV change in the nuclear electrostatic potential typically results in <1 $k_{\text{B}}T$ change in the free energy of such proteins. Hence, the direct impact of the nuclear electrostatic potential on their binding to DNA is likely to be insignificant.

Yet, it is known that these proteins are often involved in DNA-binding competition with histone octamers. For example, a recent experimental study found that the stability of nucleosomes associated with promoter regions has a strong impact on the transcription level of downstream genes, which is likely caused by DNA-binding competition between histone octamers and transcription factors (113). Thus, nucleus size-dependent changes in the nuclear electrostatic potential may have a profound effect on the balance between small DNA-binding proteins and nucleosomes by modulating stability of the latter. Such a molecular mechanism could potentially explain the connection between the nucleus size, chromatin condensation state, and the transcription level of various genes observed in many previous studies (23, 24, 25, 26, 27, 28,114).

For example, in (26) it was shown that the direct application of mechanical pressure to living cells can cause a decrease in the cell nucleus volume by ∼20−30%. Our calculations indicate that such a volume drop is associated with ∼1 mV change in the nuclear electrostatic potential, which results in ∼5 $k_{\text{B}}T$ decrease in the free energy of histone octamers assembled on DNA. As suggested by our recent study (65), this amount of energy is sufficient to tip the balance in DNA-binding competition between transcription factors and histone octamers in favor of nucleosome formation, resulting in release of transcription factors from the chromosomal DNA and global downregulation of gene transcription, both of which indeed have been observed in (26).

Furthermore, it has been previously reported that nuclei of living cells experience significant volume increase by ∼100−200% during the interphase of the cell cycle (15,115,116). Similar nuclei expansion has also been found during differentiation of human embryonic stem cells (97). According to our model, such an increase in the nucleus volume is accompanied by a strong destabilization of nucleosomes by ∼8−11 $k_{\text{B}}T$, and thus chromatin decondensation, likely leading to a higher gene transcription level, which is in good agreement with previous experimental studies (28,114,117).

**Figure 5:**
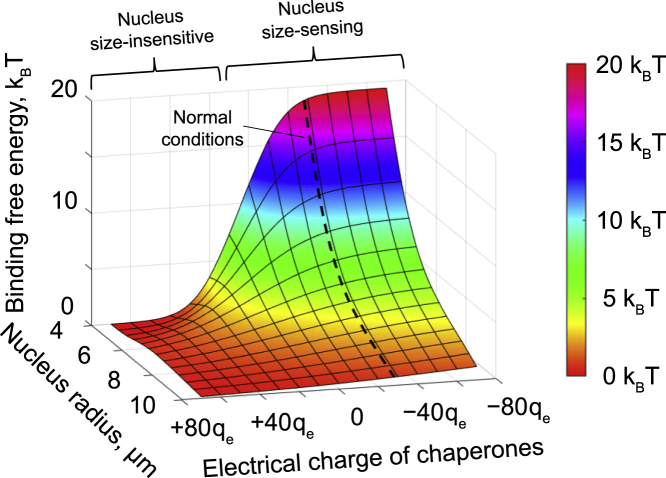
Effect of the electrical charge of chaperones on the binding free energy of histone octamers to DNA. The plot demonstrates the results of calculations based on Eq. 13, performed for various electrical charges of histone-binding chaperones. For the sake of simplicity, it was assumed in the calculations that H2A ⋅ H2B-binding chaperones (${\text{c}}_{1}$), as well as H3 ⋅ H4-binding chaperones (${\text{c}}_{2}$), have the same electrical charges: $q_{{\text{c}}_{1}\text{u}}=q_{{\text{c}}_{2}\text{u}}$. The dashed curve shown in the graph indicates the model prediction based on the electrical charges of the chaperones estimated from the nucleocytoplasmic distribution of chaperone-transported histone dimers in HeLa cells (see Online Appendix A).

Notably, our calculations suggest that the nucleus size-dependent change in nucleosome stability in fact can be quite selective. In particular, the model shows that nucleosome stability can be strongly affected by the electrical charges of histone-binding chaperones (Fig. 5), since the latter contribute to the free energy of the mobile histone-carrying protein complexes involved in nucleosome assembly. Given that there is a large number of histone-binding chaperones or chaperone-related cofactors with different electrical charges (55,118,119), living cells may be able to fine-tune the stability of nucleosomes at specific locations on the chromosomal DNA in response to nucleus size variations through selective use of histone-binding chaperones or associated cofactors.

Taken together, the results obtained in our study indicate that the nucleus size may be one of the main factors involved in the regulation of nucleosome stability through modulation of the nuclear electrostatic potential, thus contributing to the regulation of chromatin organization. In turn, since the nucleus size is determined by a tug-of-war between the osmotic pressure exerted by cytosolic macromolecules on the NE and the surface tensions of the NE and ER membrane, the model predicts the existence of a previously unknown link between chromatin organization and proteins, such as lamins, that affect physical properties of the NE or ER, in good agreement with recent experimental studies (120).

We would like to point out that, although the mean field model developed in our study does not take into account fluctuations of the nuclear electrostatic potential that may potentially contribute to multivalent interactions between biomolecules, it still provides a physically meaningful first-order approximation that highlights the critical elements involved in regulation of the nucleus size, demonstrating the importance of the latter in the regulation of nucleosome stability. Introduction of beyond mean field calculations into the developed model can further help to clarify the role of multivalent interactions in shaping chromatin structure, warranting future studies.

Finally, it should be mentioned that the model developed in our study is based on a near-equilibrium approximation of chromatin conformation and DNA-protein interactions; and, while this approximation is known to lead to a fairly accurate description of chromatin organization in living cells (30,31,33,35,37, 38, 39,41,71,72), it will be interesting to combine it with modeling of active processes that were previously suggested to affect nuclear organization, such as dynamic changes in the cell cytoskeleton or local ATP-dependent chromatin remodeling by specialized DNA-binding proteins (gyrases, helicases, etc.), to better understand their contribution to the formation and regulation of chromatin structure.

## Author contributions

L.H. and A.K.E. made the computer program used for model calculations. A.K.E. designed the research, derived formulas, carried out computations, and analyzed the data. A.K.E. and J.Y. wrote the paper.

## Supporting material

Document S1. Figures S1–S16, Tables SI, SII, and Appendices A–M

Document S2. Article plus supporting material
